# Large-Scale Channel Migration in the Sittang River Estuary

**DOI:** 10.1038/s41598-019-46300-x

**Published:** 2019-07-08

**Authors:** T. Shimozono, Y. Tajima, S. Akamatsu, Y. Matsuba, A. Kawasaki

**Affiliations:** Department of Civil Engineering, University of Tokyo, Tokyo, Japan

**Keywords:** Physical oceanography, Geomorphology

## Abstract

An estuary is a dynamic environment where marine and fluvial processes meet to form complex and transient morphology. The estuary morphology is largely determined by net sediment transport by two-way tidal flows, but the hydrodynamics also depends on the morphology of the tidal channels. The estuary inherently accommodates cyclic processes that are internally generated through hydro-morphodynamic interactions. In addition, the estuary evolves in response to changes in external forces by natural and anthropogenic factors. Morphological changes under the different controls often hinder the comprehension of the evolutionary processes of estuaries. Here we explored morphological changes in the Sittang River estuary, Myanmar, which has great morphological dynamism from extreme tidal energy and large sediment inputs, through field surveys and satellite imagery analysis. We identify an autocyclic process in a sedimentary system driving large-scale channel migration in decadal to multidecadal cycles. We show that drastic changes of the estuary morphology occasionally occur with rapid bank erosion through modulation of the cyclic channel migration under conflicting tidal and fluvial forces. This extreme case with minimal human intervention highlights channel migration as a key process in morphological evolution of tide-dominated estuaries undergoing active infilling.

## Introduction

Tide-dominated estuaries are subject to a long-term process of infilling with fluvial sediments from rivers and with marine sediments brought by asymmetric tides. Hydro-morphodynamic processes shape the supplied sediments into common landscapes: an upper channel of fluvial dominance, a central basin of high tidal energy and a funnelling basin in the lower reach. The central basin comprises blind tidal channels loosely bounded by mudflats on both sides and tidal bars in the middle, among which a main channel connects to the upper channel through a transitional segment. A tight meander usually forms in the transitional segment where the tidal force competes with the fluvial force^1^. The estuary progrades seawards if the sedimentation rate overcomes a relative sea-level rise due to climatic and/or tectonic forces. The estuary evolution, however, does not proceed monotonically, as various factors affect the sedimentary system^2–5^. Besides natural and anthropogenic effects to change external boundary conditions, the blind-channel system would evolve through simultaneous adjustment of morphology and sediment movements^6–9^.

The blind-channel system consists of ebb and flood channels that are distinguished by a dominant direction of sediment transport^10–12^. The flood and ebb channels are evasive with each other and are usually separated by tidal bars. Previous studies suggested that flood/ebb dominance varies with channel cross-section which affects the frictional damping of two-way flows^13,14^: Flood dominance characterises wide, shallow channels and ebb dominance characterises deep channels with high tidal flats. A loosely-bound channel system has a highly transient nature with formation, migration and filling of individual channels in contrast to the slow dynamics of emergent tidal channels bounded by consolidated sediments^15,16^. Continuous sediment input alters a channel cross-section to become ebb-dominant with built-up tidal flats. The resulting loss of sediment by ebb flows returns the channel cross-section to be flood-dominant. An estuary, therefore, is conjectured to balance the sediment input and output through cyclic changes in the channel system^7^. Recent experimental studies using a novel facility suggested the possibility of dynamic equilibrium with cyclic channel migration under steady tidal forcing^17–19^. However, long-term observations of subaqueous processes are limited, and the cyclic channel migration in natural tidal basins has not been well documented. A case with large-scale morphological changes and minimal human intervention can provide insights into these elusive processes.

**Figure 1:**
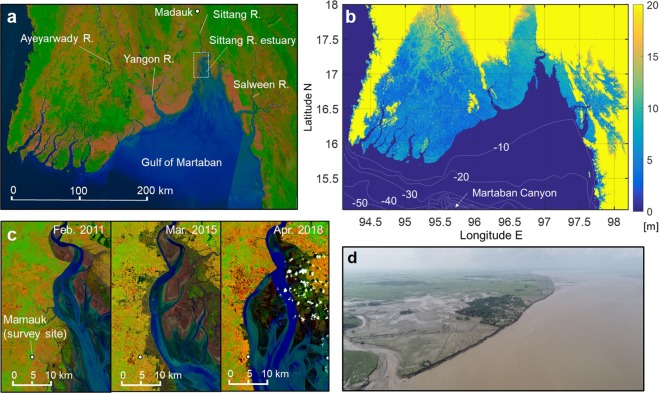
Sittang River estuary at head of Gulf of Martaban. (**a**) Map of study area on Landsat satellite image. (**b**) Topographic/Bathymetric map of study area based on Shuttle Radar Topography Mission (SRTM 1 Arc-Second Global elevation data courtesy of the U.S. Geological Survey, https://lta.cr.usgs.gov/SRTM1Arc) and General Bathymetric Chart of the Oceans data (The GEBCO Digital Atlas published by the British Oceanographic Data Centre on behalf of IOC and IHO, 2003). (**c**) Rapid bank erosion on west side of Sittang River estuary. Peak erosion rate reached as high as 3 km/yr. These images were created from Landsat imagery. (**d**) Aerial photograph of survey site on September 17^th^, 2018. Tree-planted areas are small villages of rice farmers, and one in centre, Mamauk, was being abandoned due to severe erosion at time of survey. Landsat imagery courtesy of NASA Goddard Space Flight Center and U.S. Geological Survey. Maps were created using MATLAB ver. R2006 from Mathworks Inc. Figure labels were added using Microsoft Powerpoint 2016.

We found an ideal case for this study in the Sittang River estuary, at the head of the Gulf of Martaban, Myanmar. The Gulf of Martaban is a typical funnel-shaped basin that opens widely to the Andaman Sea with its head bounded on the east by a rocky scarp (Fig. 1a,b). The landward offset of the coastline from the west side is attributed to tidal force variation over uneven shelf bathymetry^20^. The semidiurnal tides coming from the Indian Ocean have large amplitude in shallow areas of the Andaman Sea^21^ and further intensify into the gulf due to strong funneling effect. The tidal range inside the gulf varies from 3 m to 7 m over the course of spring-neap cycles^22^. Three major rivers—the Ayeyarwady, Sittang and Salween—supply a large amount of fluvial sediments into the ocean (Fig. 1a). The total amount of sediment output through the rivers and their distributaries was roughly estimated as 370–600 Mt/yr^23^. A significant proportion of fine-grained sediments continuously enter the head of the gulf by flood-dominant tides. The high convergence of sediment fluxes maintains the great dynamism of the estuary morphology.

Rapid bank erosion has occurred recently on the west side of the estuary. The erosion rate reached as high as 3 km/yr in some places, and small villages of rice farmers adjacent to the estuary have been gradually abandoned (Fig. 1c,d). With no effective measure against the irresistible force of nature, coastal residents who had resided there for decades were forced to move inland. Facing the central basin of the estuary, the area being eroded is where flood tides form a bore front during spring tides. Toward the upper reach of the estuary, the excessive tidal energy rapidly dissipates through bore-induced turbulence and by high bed friction over the mudflats. The energetic bore in the gulf has long been recognized^24,25^, but its characteristics and impacts on morphological changes are still largely unknown. Extreme flood flows are generated by highly asymmetric tides in bore-tidal estuaries^26^. The rapid erosion is evidently due to actions of the intense tidal flows which dominate riverine flows at this downstream site of the Sittang River. Additional factors modulating the tidal force along the bank must be sought in order to explain the extreme short-term bank erosion. These could be due to natural and/or anthropogenic changes in external forces, or otherwise be cyclic processes of tidal channels that were internally generated in the estuary. We studied the estuary evolution in different time scales through field and satellite observations to address the issue.

## Results

### Long-term estuary evolution

The coastlines around the Gulf of Martaban and along the Ayeyarwady Delta have been surprisingly stable on a centennial time scale despite large sediment inputs from the rivers^27^, which were quickly dispersed into the ocean by strong tidal flows without contributing to coastline progradation^28^. The gulf acts as a large sediment sink with the Martaban Canyon (Fig. 1b) as a sediment drain to the Andaman Sea^20^. The Sittang River estuary is an exceptional area, where coastlines exhibit significant variations on decadal to multidecadal time scales. In order to observe the estuary evolution over past decades, we extracted coastlines around the estuary from Landsat imagery for the years 1973 to 2017. The coast here is defined as the seaward margin of vegetation neglecting lower mudflats that are highly dependent on semidiurnal tidal water-level changes. To observe an even longer trend, we derived additional coastline data from two old maps compiled in the 1940s and the mid 19th century (see Supplementary Fig. S1 for the original maps).

**Figure 2:**
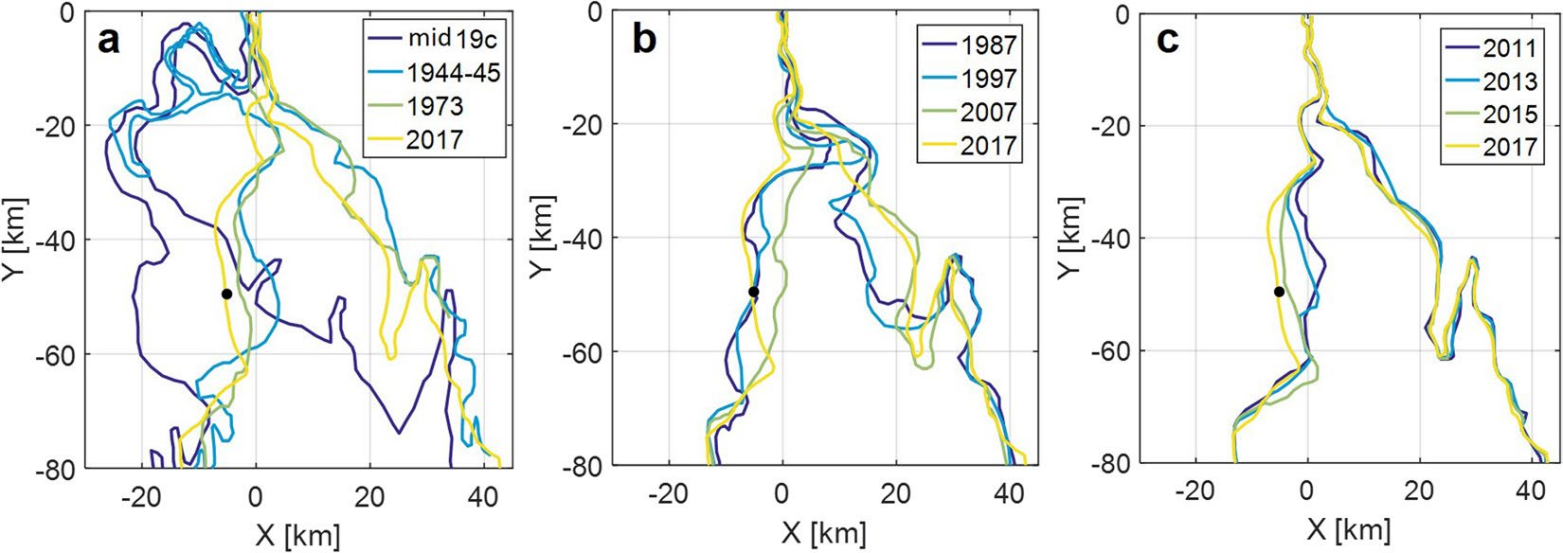
Evolution of Sittang River estuary on different time scales. These coastlines represent vegetation lines extracted from Landsat imagery. Two old coastlines (1940’s and mid 19^th^ century) were derived from old printed maps. Black dot indicates survey site at Mamauk. Supplememtary Fig. S2 provides comparisons of two successive coastlines for each figure. (**a**) Centennial changes. (**b**) Multidecadal changes. (**c**) Intradecadal changes. Figures were created using MATAB ver. R2006b from Mathworks Inc.

The central basin of the estuary has been greatly displaced since the 19th century (Fig. 2a), when the upper channel of the estuary mostly meandered westward. At that time, the central basin was ~20 km west of its present location, but its shape and area were similar to the present-day basin. The central basin moved to the present location probably due to the meandering channel being cut off from the late 19th to early 20th century, and the old channel remained as an abandoned channel (Supplementary Fig. S1). The upper channel has been filled and stabilised along the rocky scarp on the east side and has become straighter since then. From the 1940s to the present, the central basin has moved slightly back to the west, while the upper channel has been further narrowed. In contrast, coastlines in the lower reach of the estuary have experienced relatively small changes over the same long period.

The multidecadal coastline changes also exhibit apparent cyclic and smaller magnitude lateral displacement of the central basin (Fig. 2b). A tight meander was present in the transitional segment in the 1990s, but it was rapidly eroded away in the early 2000s. The loss of the tight meander shifted the central basin eastward by 2007. An erosional trend has started on the west side since then. The coastline changes in the past decade show rapid coastal erosion extending from the upper to lower reach (Fig. 2c). Satellite images suggest that a large portion of the eroded sediments was deposited on the east side of the estuary (Fig. 1c). The rapid erosion appears to be part of the cyclic displacement of the central basin in view of the long-term morphological changes. The central basin has widened in recent years, as bank accretion on the east side is a slower process.

The rapid bank erosion cannot be linked directly to changes in external forces affecting the estuary. We extracted a time series for the bank position near the small village of Mamauk (Fig. 1c) for the years 1987 to 2017. The erosional trend started in 2013 after a decadal trend of large bank accretion (Supplementary Fig. S3a). Daily observed water-level and flow discharge data are available during the same period from Madauk Station, which is 60 km upstream from the mouth of the Sittang River (Fig. 1a). The riverine flow has a typical monsoon seasonality, the peak water level being observed every August with a flow discharge of 3000–5000 m^3^/s (Supplementary Fig. S3b,c). Despite the construction of small reservoirs on their tributaries, the major rivers flowing to the gulf have been less controlled by damming than have other Asian rivers^20^. Annual maximum and average flow discharge show no obvious trend, whereas dry-season discharge exhibits a slight increase over the period. While the increase in low flow discharge potentially affects the estuary morphology in a long term, there is no obvious trend in fluvial factors that can be correlated with the recent bank erosion. Some estuaries reportedly respond to long-term tidal variations such as the 18.6-year lunar nodal cycle^29,30^. However, the decadal tide component, which is small relative to 4.4-year tidal modulation by the lunar perigee cycle in this region^31^, does not account for the rapid morphological changes either (Supplementary Fig. 3a). Without possible external causes, we hypothesize that the rapid bank erosion occurred as a consequence of large-scale channel migration driven by an internally generated process.

### Rapid bank erosion

**Figure 3:**
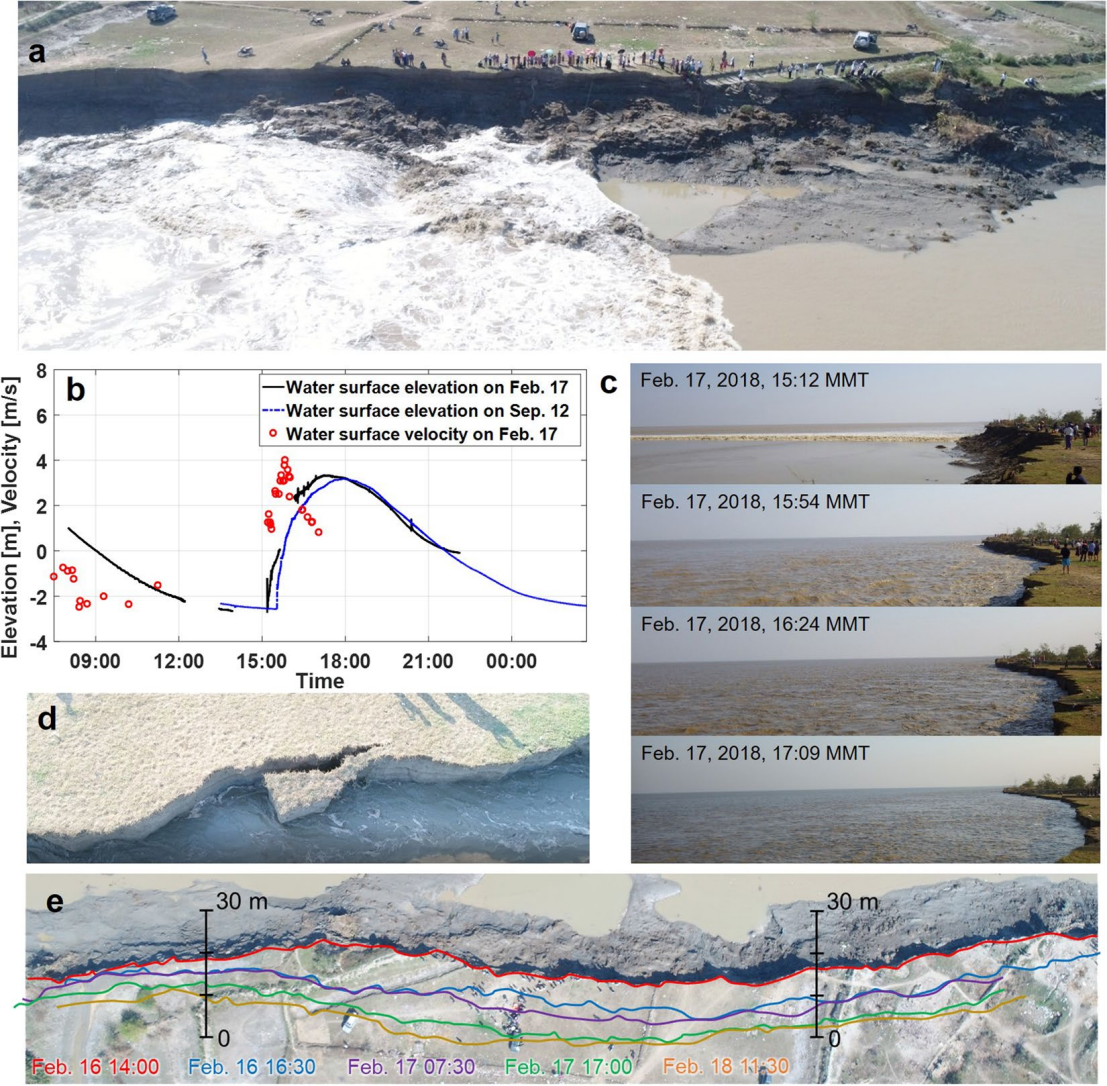
Rapid bank erosion by intense tidal flows. (**a**) Aerial view of survey site fronted by eroded bank upon tidal-bore arrival. (**b**) Observed water-level and flow-velocity changes over spring tidal cycle in dry and rainy seasons (Water-level profile in dry season has some data breaks). (**c**) Bank erosion process during flood phase from dry-season survey. (**d**) Top view of the cantilever bank failure: overhang block is collapsing. (**e**) Bank line retreating 10–20 m over four tidal cycles. Graphs were created using MATAB ver. R2006b from Mathworks Inc. Figure labels were added using Microsoft Powerpoint 2016.

We conducted field observations of the erosional process on the west side of the estuary. A survey site was set near the small village of Mamauk (Fig. 1c), for accessibility in both dry and wet seasons. The site is fronted by a steep bank, which is the result from severe erosion (Fig. 3a). The top of the bank is very flat on an elevation similar to that of the highest tide water. The bank consists almost entirely of fine-grained, cohesive sediments, and the cut bank face reveals horizontal layers several millimetres thick indicating that the flatland was formed by gradual mud accumulation (Supplementary Fig. S4). We carried out the first survey during the dry season in 2018, from February 16–18, with the primary objective of investigating tide characteristics and the bank-erosion process. We performed a follow-up survey in 2018 to observe fluvial influences in the rainy season, during September 12–13. In both surveys, we measured water-level changes due to a spring tide, and shot videos of tidal flows and bank-erosion processes both from the ground and using an unmanned aerial vehicle (UAV). In the dry-season survey, we also obtained water-surface velocity data near the bank by tracking floating objects and measured the bank line several times during the survey period.

The observed tidal-wave profiles highlight the notably asymmetric features of the spring tides in the central basin (Fig. 3b). The water level rapidly rose after the bore arrival at the lowest tide and after two hours reached the highest level, ~6 m above the lowest water level. The turbulent bore had a height of 0.5–0.8 m, arrived with a rumble noise and instantly reversed a weak ebb flow to become a strong flood flow (Supplementary Video S1), which rapidly developed into a destructive flow with a speed >4 m/s. The high flood flows were sustained for a short time interval and then rapidly decelerated toward high tide. The water surface gradually receded from the highest level, and the tidal flow turned into a longer ebb phase. There was no significant difference in the observed profiles between dry and rainy seasons, implying that the seasonally changing fluvial factors did not remarkably change spring tide amplitude at this downstream site.

We took sequential photographs of the bank erosion process during the flood tidal phase (Fig. 3c). The bore front did not significantly erode the bank, although it violently hit against the lower bank. Bank erosion mostly occurred during the short interval of the peak flood flow in a cantilever failure mode, which is commonly observed in cohesive riverbank erosion^32^. The destructive flow eroded a lower part of the bank to produce overhanging blocks (Fig. 3d), and we observed blocks of several meters in length repeatedly collapsed down into the flowing water (Supplementary Video S2). The bank collapse ceased before high tide, and it was seldom observed during the ebb phase. We measured bank lines over the course of four tidal cycles and superposed them on a top-view image of the initial bank (Fig. 3e). Because the slump blocks at the foot of the bank prevented lower bank erosion^33^, a large bank retreat occurred only every two tidal cycles. On average, the bank eroded 3–5 m per spring tidal cycle, but we found that the bank line had moved ~500 m inland when we revisited the site in the rainy season after seven months. These erosion rates suggest that bank erosion intensely progressed by the impulsive flood flows associated with spring tides. Additionally, a satellite observation of annual coastline changes for the years 2016 to 2017 shows that the bank erosion proceeded at an almost constant rate over dry and rainy seasons (Supplementary Fig. S5).

### Cyclic channel migration

**Figure 4:**
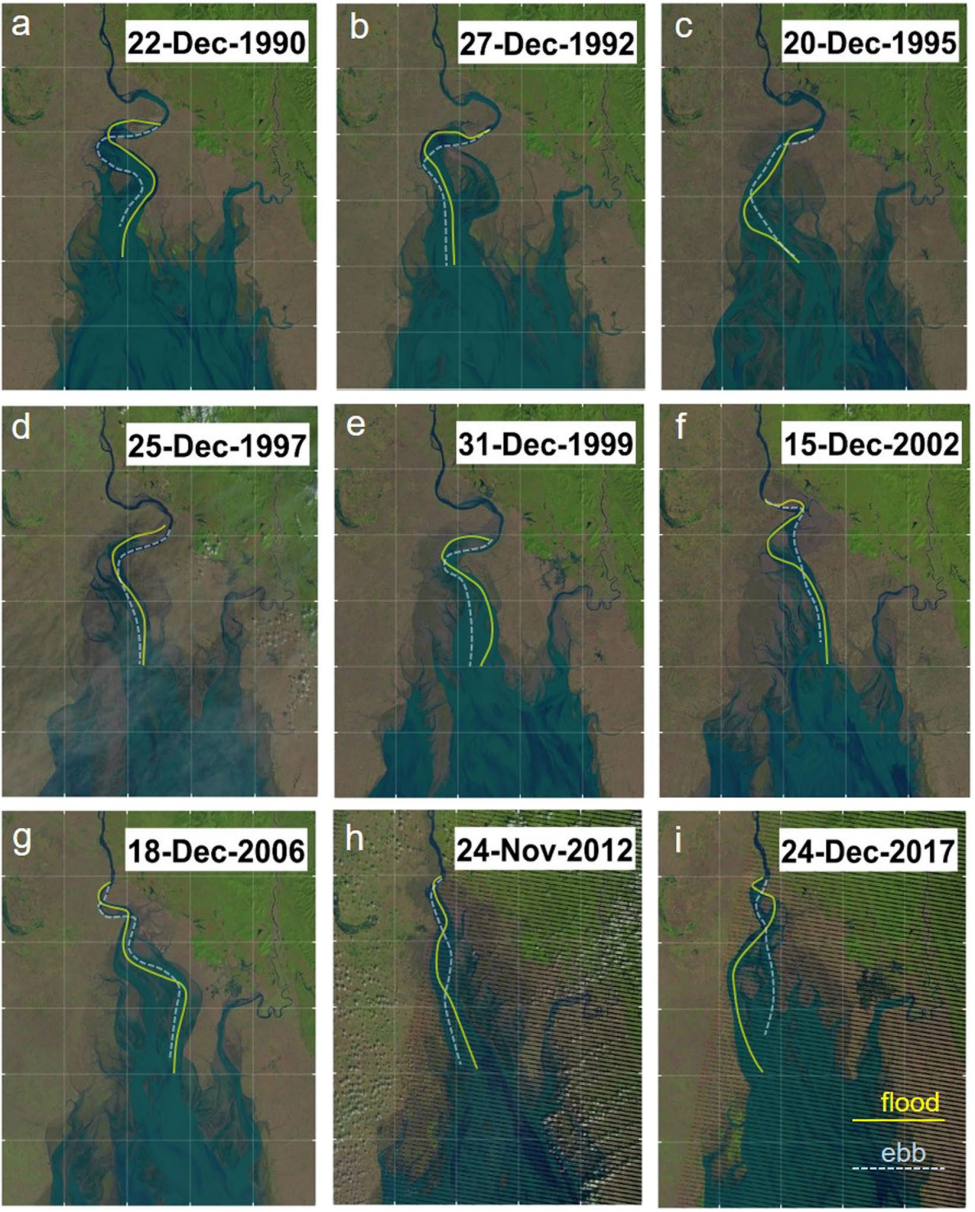
Cyclic Channel migration from 1990 to 2017. Cyclic channel migration is illustrated by sequential Landsat images at every quarter phase of the cycle. Yellow and light-blue lines highlight dominant flood and ebb flow courses in main channel, respectively. Sinuous channel migrated laterally with its end fixed in upper channel. Channel migration caused erosion on two sides of central basin alternately. Landsat imagery courtesy of NASA Goddard Space Flight Center and U.S. Geological Survey. Figure was created using MATAB ver. R2006b from Mathworks Inc. Figure labels were added using Microsoft Powerpoint 2016.

We observed the evolution of tidal channels in the central basin using the Landsat imagery for the years 1987 to 2017. Some high-quality images taken during low tides clearly display blind-channel geometry. A sinuous channel usually forms about the estuary axis in the north-northwest direction, which connects to the upper, straight channel through a meandering segment of the tidal–fluvial transition. The sequential images over the 31 years illustrate that the tidal channel has migrated like an oscillating string in a stationary wave mode with a fixed end in the upper channel (Supplementary Video S3). To illustrate the cyclic channel migration, we extracted nine satellite images at every quarter phase of the cycle and highlighted the main channel with dominant flood and ebb flow courses inferred from tidal bar and cuspate bank forms (Fig. 4). One whole cycle is identified from 1990 to 1999 with a period of approximately 9 years. Subsequently, the tight meander in the transitional segment was rapidly eroded away and evolved into a relatively loose meander of cuspate form from 2000 to 2003. This appears to have introduced a significant irregularity to the cycle, and the following channel migration occurred with nearly double amplitude and period. The cyclic channel migration induces bank erosion alternately on the two sides of the central basin, and the migration amplitude has peaked in the range of the central basin where we observed the rapid bank erosion. The cyclicity is maintained by active sediment accretion opposite the active bank erosion, which keeps the basin width smaller than the migration amplitude.

**Figure 5:**
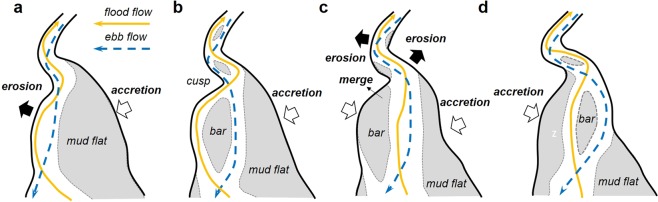
Channel-migration subprocesses. Lateral migration of tidal channel proceeds through meeting/parting of flood and ebb channels, which are evasive with each other. (**a**) Flood and ebb flows form dominant channel on alternate sides, and rapid bank erosion occurs during this phase. (**b**) Channel meanders during bank erosion. Ebb flows no longer follow it from opposite side and thus create new, separate channel. (**c**) Flood flows shift to new channel as ebb flows deepen it. Channel along bank is filled out without active ebb flows. (**d**) Flood and ebb flows separate from each other as meander channel migration proceeds. Subsequently, flood flows shift to ebb channel to form dominant channel along other side of central basin. Illustrations were created using Microsoft Powerpoint 2016.

Further detailed observation of the flood–ebb channel evolution reveals cyclic subprocesses causing channel migration. Flood tides produce high flow velocity for a short period of time, while ebb tides are sustained for a longer period with lower velocity. Based on the field and satellite observations, they appear to play different leading roles affecting the channel geometry. Flood tides that decay upward lead the meander channel migration through bank erosion, whereas ebb tides excavate a deep channel by concentrating in a narrow zone. Channel-migration subprocesses are illustrated in Fig. 5. Bank erosion is active when flood and ebb flows form a dominant channel on one side of the central basin (Fig. 5a). As high flood flows produce a concave bank with an upper depositional bar, ebb flows are no longer able to follow the same flow course, which results in the formation of a new ebb channel on the other side and tidal bars between the two separated channels (Fig. 5b). Because of weakening of the ebb flows, the channel along the bank increases flood dominance and is gradually filled in by residual sediment flux from the lower reach. Subsequently, flood and ebb flows develop a dominant channel in the centre of the estuary (Fig. 5c) and again, the flood channel undergoes meander migration and the ebb channel parts from it (Fig. 5d). Finally, the main channel develops on the other side of the central basin inducing the bank erosion, and then, initiates the opposite processes returning to the original side. The main channel cyclically migrates from one side to the other side of the central basin through the mutually evasive evolution of flood and ebb channels. The autocyclicity appears to be maintained by impulsive flood flows continuously giving perturbation to the ebb flow course through bank erosion and deposition in the upper confined basin.

**Figure 6:**
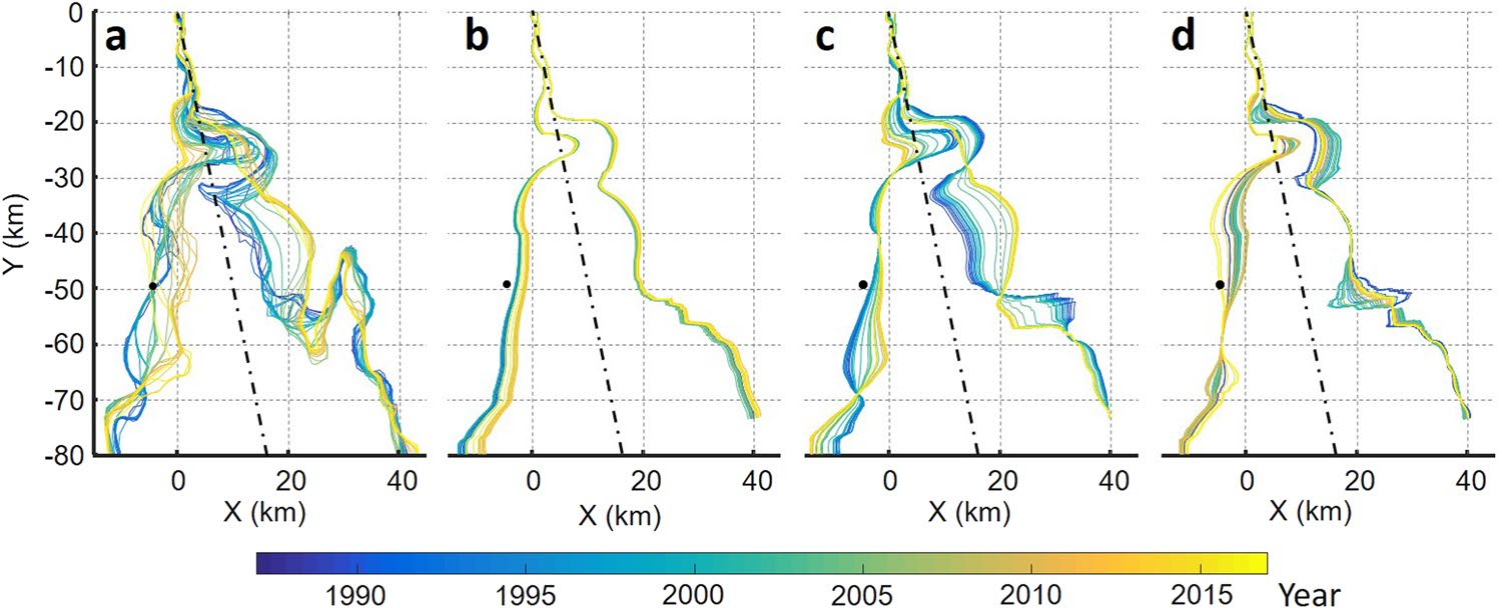
Empirical orthogonal function (EOF) decomposition of coastline changes from 1987 to 2017. Coastline changes deconstructed into orthogonal components. Black dash–dot lines represent estuary axis used in EOF decomposition. (**a**) Extracted coastlines for years 1987 to 2017. (**b**) First-mode EOF variance. Base coastline was created by averaging these coastlines over period. (**c**) Second-mode EOF variance on base coastline. (**d**) Third-mode EOF variance on base coastline. Second- and third-mode variances account for 81–85% of total variances excluding first-mode variance. Figure was created using MATAB version R2006b from Mathworks Inc.

Estuary evolution is complex, involving multiple factors. Therefore, we applied an empirical orthogonal function (EOF) analysis to the coastline dataset for the years 1987 to 2017, during all of which high-quality satellite images were available (Fig. 6a). We deconstructed the estuary morphology into primary (orthogonal) components. The first three modes of the deconstruction account for over 99% of the total variance, while a lower-mode variance represents longer-term and more-global trends in the morphological changes. The first-mode variance represents averaged forms of the estuary (Fig. 6b), showing a slight progradation in the lower portion of the coastline on the west side but no significant change in the inner estuary over the three decades. It is worth noting that the progradation of the west bank has a significant correlation with the increasing trend of dry-season river discharge (*R* = 0.62, see Supplementary Fig. S3). The second- and third-mode variances are plotted on a base for which the coastline was created by averaging the first-mode variance over the 31-year period (Fig. 6c,d). The second-mode variance depicts the drastic transformation of the estuary through the loss of the tight meander in the early 2000s, in which the transitional segment shifted westward, while the central basin expanded eastward. The recent erosional trend on the west coast was deconstructed as the third-mode variance, which becomes significant only after the early 2000s showing a strong link to the second-mode variance.

The rapid shift of the transitional segment in the early 2000s appears to have been a turning point for morphological changes in the estuary. The subsequent satellite images depict a westward shift of the channel migration with increasing amplitude (Fig. 4). The recent erosional trend on the west coast can be attributed to the modulation of the cyclic channel migration by the rapid shift from tight to cuspate meanders in the transitional segment. A lack of satellite images in the early 1980s does not allow us to observe the tight meander formation. Nonetheless, satellite images in the 1970s suggest that the tight meander developed from the cuspate meander (Fig. 2a), which further implies that the tightening of the transitional segment is a recurrent process under mixed fluvial–tidal control. The tight meander probably grows to resolve the conflict of the upper and lower channel migration under different controls, and it undergoes rapid erosion at a critical stage. This process would have a certain irregularity associated with variations of the fluvial and tidal forces by natural and anthropogenic factors.

## Discussion

This study highlighted the cyclic channel migration as a key process in estuary evolution, which triggers alternating erosion and accretion on opposing sides of the estuary in decadal to bidecadal cycles. The cyclic channel migration occurs through the mutually evasive evolution of ebb and flood channels, but the cause of the cyclicity remains unclear as to why this estuary has the tremendous cycle that others do not. The asymmetric tides of large amplitude may be a key factor, which possibly lead to the distinct roles of flood and ebb flows on estuary morphology and thereby maintain the cyclicity of the channel system. However, other estuaries in similar tidal settings, such as Qiantang estuary in China, do not exhibit an obvious cycle^34^. Another peculiar feature of this estuary is presence of large sediment sources on the sea side, originated from the Ayeyarwady and Salween. The continuous sediment supply is likely to support the fast bank accretion on the opposite side of the bank erosion. Further studies will be required to address this issue with sufficient data support.

The estuary yields an equilibrium state with the channel migration cycle through which residual sediment transport in and out of the estuary is roughly balanced. However, the channel migration is occasionally modulated by morphological changes in the transitional segment under a mixed tidal–fluvial control. The pivotal segment probably evolves in an irregular cycle between a cuspate and tight meander with a changing balance of tidal and fluvial forces. This multi-decadal cycle can also be viewed as the depositional-erosional cycle of estuary evolution. Both the upper channel and the central basin are filled with a tight meander, while active channel migration sweeps sediments out from the central basin with a cuspate meander. The estuary infilling likely proceeds with the downshift of the transitional segment through the destruction and creation of tight meanders.

## Methods

We produced the coastline dataset from Landsat satellite imagery from the years 1973 to 1978 and 1987 to 2017. Cloud-free satellite images of the estuary in November to December were obtained using Earth Explorer by the U.S. Geological Survey. We extracted vegetation lines on both sides of the estuary neglecting lower mudflats and small-scale morphological features such as narrow creeks and isolated tidal bars, which vary on a short time scale. The detection error was found to be within a few hundred meters when it was evaluated on some stable portions of coastline. A major uncertainty arises from an ambiguity of coastline definition in a depositional phase. Coastlines under the definition above occasionally move a few kilometers seaward over one year when an extensive mud flat rises above the high water level. Therefore, the bank accretion process is more or less represented by step-like changes on the time axis. This poses an uncertainty for an instantaneous estuary planform when the boundary is not clear. Nevertheless, we could capture global trends of the estuary evolution with low-order EOF variances, since local changes associated with the uncertainty are decomposed into higher-order EOF variances.

The two additional coastlines in the 1940s and the 19th century were derived from two old maps: a 1:250,000-scale map by the US Army Corps of Engineers (U542, NE47-13) based on surveys from 1944–45 and a topographic map of Lower Sittang Valley that appeared in an 1876 geography book^35^. The survey period of the latter old map was not given; we assumed it to have been the mid 19th century. Supplementary Fig. S1 shows these original maps together with another map in the late 19th century. Surveying errors are inherent in the oldest map, but topographic changes afterwards were traceable on later period maps from different sources. Therefore, we employed the coastline from the oldest map, given large topographic changes relative to the possible error. These two maps were rectified so that city locations and stable landforms on the maps agree with those on the satellite images.

For the EOF analysis, we introduced a longitudinal coordinate along the estuary axis and resampled the coastline data on the west and east as displacements from the axis at 30 m intervals. Then, we stored the 31-year dataset on each side of the estuary in a matrix arranging each coastline into a row vector and deconstructed it into orthogonal components by the eigenvalue expansion. The EOF deconstruction was implemented independently for the two sides with a simple Matlab algorithm^36^. Coastline displacements were not demeaned prior to the EOF deconstruction, and thus, the first-mode variance represents the mean coastline which resembles the statistical mean^37^. The base coastline was created by averaging the first-mode coastlines over the entire period, and the second- and third-mode variances were displayed from it in Fig. 6.

We measured the water surface, flow velocity and bank displacement over tidal cycles in the field surveys. The water-level measurement was performed with a bottom pressure sensor fixed on a muddy bed by a wooden pile during low tides. The sensor was collected after one day since the bank retreated quickly over tidal cycles. The bottom pressure sensor captured water-surface variation over a full tidal cycle, although short data breaks occurred due to sensor exposure during low tide and oscillations by turbulent flows during flood tide in the dry-season data. Flow-velocity measurement was done by tracking floating objects away from the bank where flows were not directly affected by bank-generated vortices. For the bank-line measurement, we took orthogonal images of the bank line with ground-control points using an UAV and detected the bank lines from the rectified aerial images.

## Supplementary information

Supplementary Figures

Tidal bore arrival at the survey site

Rapid bank erosion in a cantilever failure mode

Sequential satellite images of the Sittang River estuary from 1987 to 2017. Landsat imagery courtesy of NASA Goddard Space Flight Center and U.S. Geological Survey.

## Data Availability

The datasets generated during this study are available from the corresponding author on reasonable request.
