# Supplementary material for: Large-Scale Channel Migration in the Sittang River Estuary
Source: Sci Rep. 2019 Jul 8;9:9862. doi: 10.1038/s41598-019-46300-x (PMC6614478; doi:10.1038/s41598-019-46300-x)
Supplement: Supplementary file 1 — Supplementary Figures [file 41598_2019_46300_MOESM1_ESM.pdf]

Supplementary Figures

Titile: Large-Scale Channel Migration in the Sittang River Estuary

Authors: T. Shimozono[1*], Y. Tajima[1], S. Akamatsu[1], Y. Matsuba[1] and A. Kawasaki[1]

[1]Department of Civil Engineering, University of Tokyo, Tokyo, Japan.

*Corresponding author: Takenori Shimozono (shimozono@coastal.t.u-tokyo.ac.jp)

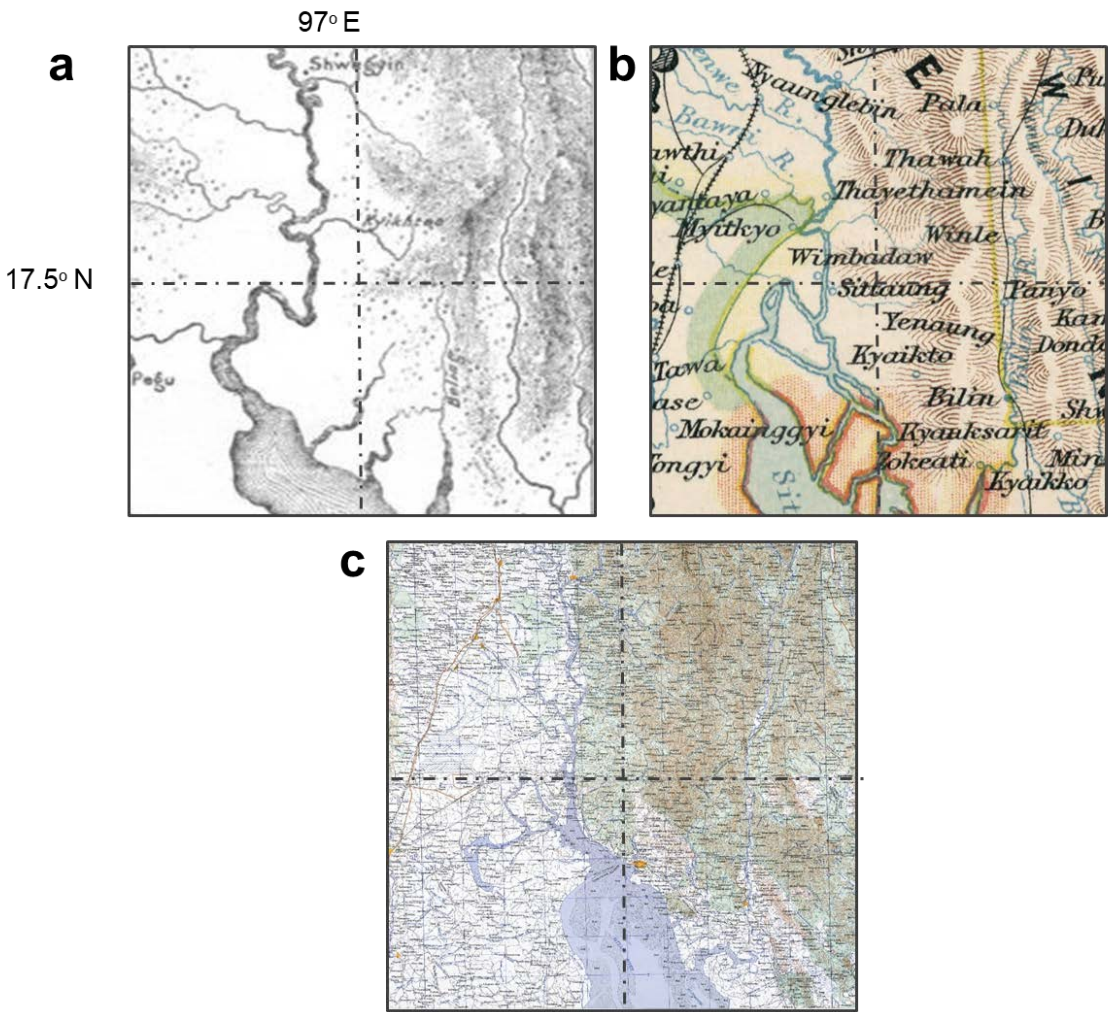

Supplementary Figure S1 | Old maps of Sittang River estuary cropped from different map sources. **a,** mid 19c century. **b,** late 19c century. **c,** 1940s. Details of map sources are listed below.

a: Lower Sittang Valley (scale 1:2,000,000, survey period: unknown)
Reclus, E. (1876). The universal geography: earth and its inhabitants, Vol. 8, 454p, London: J.S. Virtue & Co., Limited
https://archive.org/details/universalgeograp08recl/page/454
b: British Burma (South), Andaman, and Nicobar Islds.
(scale 1:3,225,000, survey period: unknown)
Sir W.W. Hunter, K.C.S.I. (1894). Atlas of India: containing sixteen maps & complete index, with an introduction, W. & A. K. Johnston. Edinburgh & London.
c: U.S. Army Map Series U542, NE47-9 (Pegu)
(scale 1:500,000, survey period: 1941, 1945-46)
Printed by the Army Map Service, Corps of Engineer in 1954
http://legacy.lib.utexas.edu/maps/ams/burma/txu-oclc-6924198-ne47-9.jpg

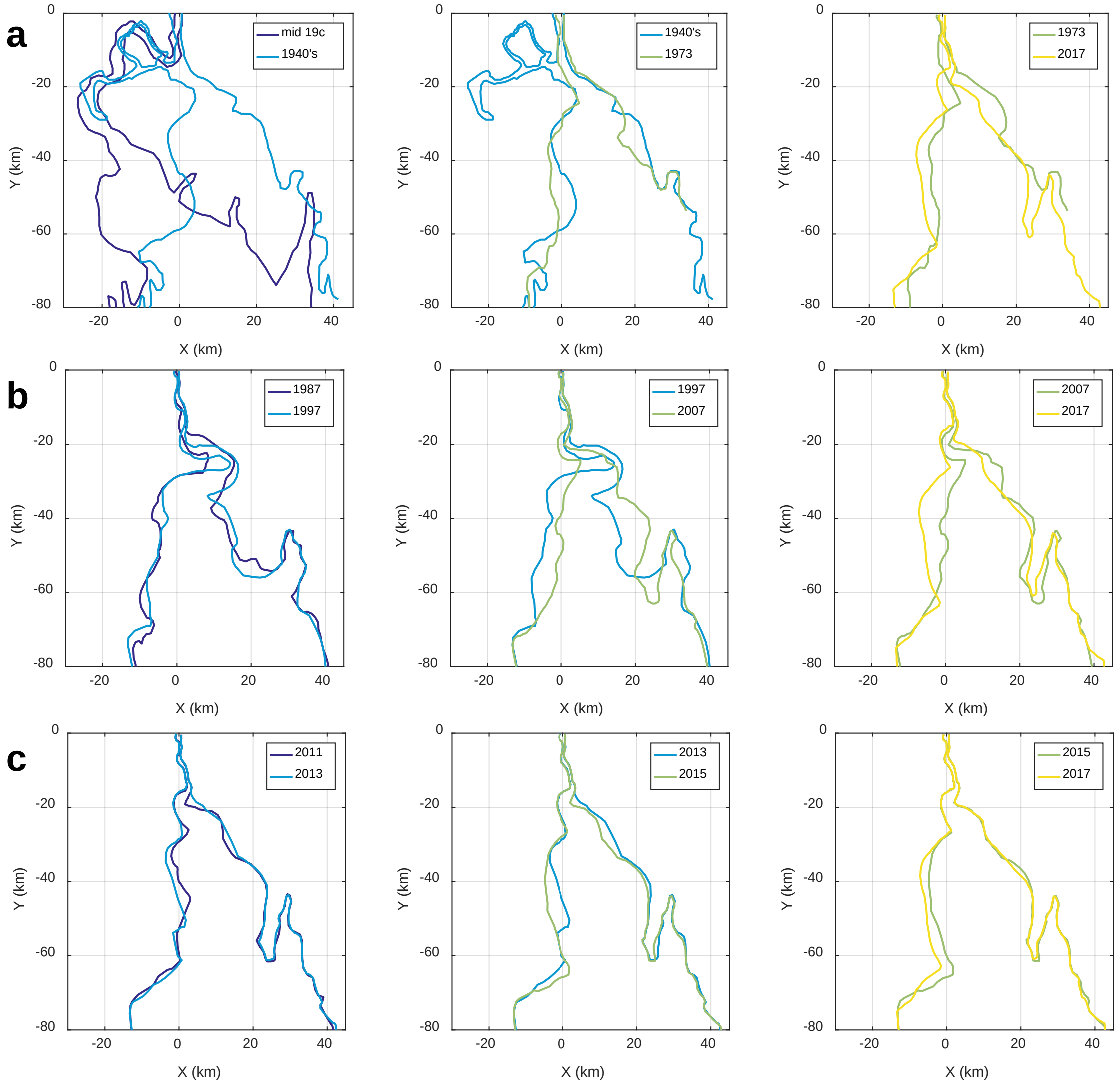

Supplementary Figure S2 | **Evolution of Sittang River estuary on different time scales**. Comparisons of two successive coastlines for same dataset in Fig. 3. **a**, Centennial changes. **b**, Multidecadal changes. **c**, Intradecadal changes.

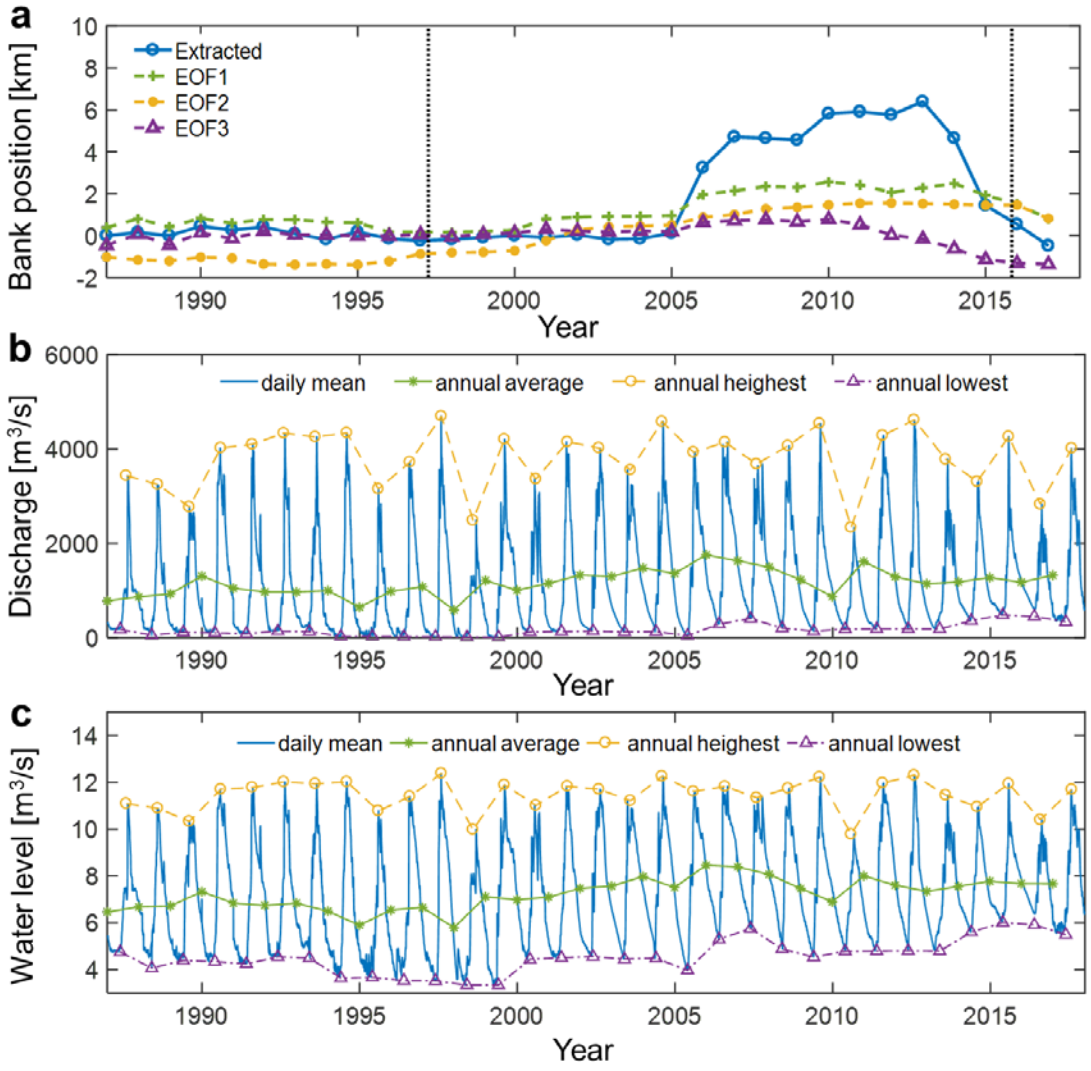

Supplementary Figure S3 | **Bank position near Mamauk and daily observed data in lower Sittang River for years 1987–2018**. **a,** Time series of bank position near small village of Mamauk extracted from Landsat imagery and associated EOF deconstruction. Vertical dotted lines indicate timings of maximum tide amplitude by 18.6 yr tidal modulation. **b,** Daily mean discharge. **c,** Daily mean water level. Daily mean discharge was estimated from daily mean water level. Data were observed at Madauk station (~60 km upstream from river mouth).

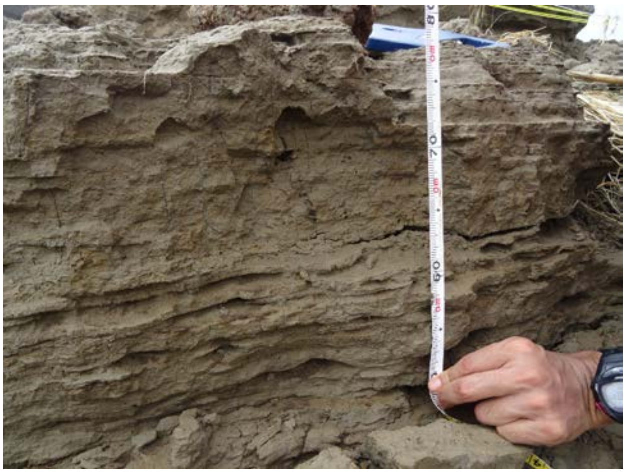

Supplementary Figure S4 | Cohesive bank cut. Bank-cut surface exposes thin layers indicating that high land was created by mud accumulation over neap–spring tide cycles.

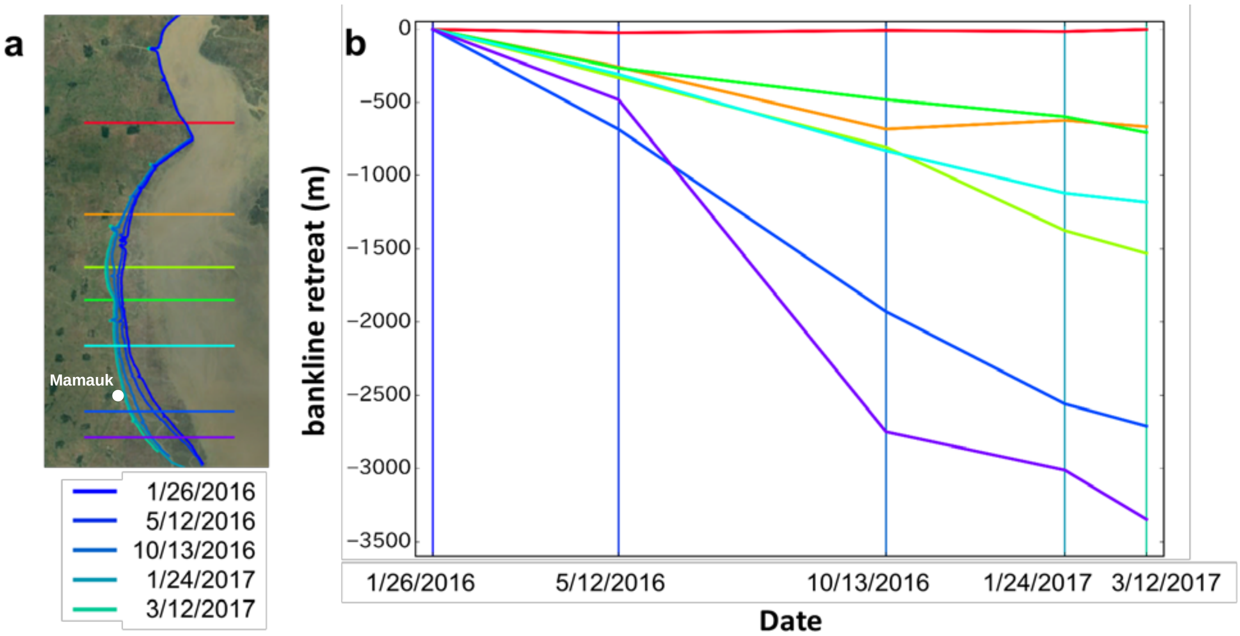

Supplementary Figure S5 | Annual changes of west coastline from January 2016 to March 2017. a, Coastlines on west side of Sittang River estuary extracted from Synthetic Aperture Radar (SAR) imagery by ALOS2(©JAXA) at five different timings. **b,** Bank retreat plotted against time for seven transects shown with same line colours as in **a**.
